# Arrested Pneumatization of the Sphenoid Sinus on Large Field-of-View Cone Beam Computed Tomography Studies

**DOI:** 10.3390/dj3020067

**Published:** 2015-05-11

**Authors:** Mehrnaz Tahmasbi-Arashlow, Sevin Barghan, Jeffrey Bennett, Rujuta A. Katkar, Madhu K. Nair

**Affiliations:** 1 Oral and Maxillofacial Radiology, University of Florida College of Dentistry, 1395 Center Dr, Room D8-6, Gainesville, FL 32610, USA; E-Mails: MTahmasbiArashlow@dental.ufl.edu (M.T.-A.); sbarghan@dental.ufl.edu (S.B); rkatkar@dental.ufl.edu (R.A.K.); nairmk@radiology.ufl.edu (M.K.N.); 2 Neuroradiology, College of Medicine, University of Florida, Post Office Box 100374, Gainesville, FL 32610-0374, USA; E-Mail: bennja@radiology.ufl.edu

**Keywords:** arrested pneumatization, sphenoid sinus, development, craniofacial lesion, Cone-Beam Computed Tomography

## Abstract

Arrested pneumatization of the sphenoid sinus is a normal anatomical variant. The aim of this report is to define cone beam computed tomography (CBCT) characteristics of arrested pneumatization of sphenoid sinus in an effort to help differentiate it from invasive or lytic skull base lesions. Two cases are presented with incidental findings. Both studies, acquired for other diagnostic purposes, demonstrated unique osseous patterns that were eventually deemed to be anatomic variations in the absence of clinical signs and symptoms although the pattern of bone loss and remodeling was diagnosed as pneumatization of the sphenoid sinus by a panel of medical and maxillofacial radiologists following contrasted advanced imaging. It is important to differentiate arrested pneumatization of the sphenoid sinus from lesions, such as arachnoid granulations, acoustic neuroma, glioma, metastatic lesions, meningioma, or chordoma, to prevent unnecessary biopsies or exploratory surgeries that would consequently reduce treatment costs and alleviate anxiety in patients.

## 1. Introduction

With the advent of cone beam computed tomography in dentistry, use of large field of view studies is fast becoming the standard of care in orthodontics, dentofacial orthopedics and implant dentistry. Since the appearance of relatively lucent lesions mimic osteolysis in the skull base captured on such studies and trigger additional investigations, it is imperative that benign conditions noted in such studies be considered in the differential diagnoses prior to further referral and advanced imaging. This report presents two cases in patients reporting for orthodontic or orthoganthic surgery and dental implant treatment, in whom lytic lesions were investigated owing to extensive effacement of the sphenoid bone architecture.

The process of pneumatization of the paranasal sinuses and skull base start in utero and develops through young adulthood [1,2]. The sphenoid sinus develops within the sphenoid bone, with varying degrees of pneumatization that do not appear overly lytic on imaging studies [3]. Sphenoid contains red bone marrow (hematopoietic tissue) without air at birth and this continues until the age of four months [1,4]. The hematopoietic tissue is replaced slowly by non-hematopoietic mesenchymal cells known as yellow or fatty bone marrow [1,5]. Causes of this conversion are not fully understood, though, vascularity, temperature and low oxygen tension may play a part [5]. Conversion of red to yellow bone marrow occurs by the age of two years in most individuals [1,4], leading to the ingress of epithelial cells to form respiratory mucosa [6]. In general, maturation of sphenoid aeration is completed by the age of 12 to 14 years [7]; however, aeration continues to occur through the end of the third decade of life [2]. For unknown reasons, the pneumatization process can sometimes be interrupted [8]. This phenomenon causes appearance of a radiographic feature referred to as arrested pneumatization of the sphenoid sinus, which is a normal anatomic variant [1], and usually discovered incidentally during imaging of the skull base [3]. Patients are essentially asymptomatic [3]. Arrested pneumatization being uncommon can lead to misinterpretation of the signals as skull base pathoses, necessitating unnecessary imaging and interventional procedures [1].

This report presents two cases of arrested pneumatization of the sphenoid sinus as seen on large field-of-view (FOV) cone beam computed tomography studies (CBCT) acquired for orthodontic/maxillofacial surgical diagnostic tasks and dental implant placement procedure. The aim of the report is to define CBCT characteristics of arrested pneumatization of sphenoid sinus in an effort to help differentiate it from aggressive, invasive skull base lesions. CBCT is suboptimal for interpretation of soft tissue entities and spatial resolution is significantly higher than that of multi-detector computed tomography (MDCT). Spatial resolution is better than MDCT by an order of magnitude but the modality is best suited for imaging osseous and dental tissues. Image acquisition is completed in 20 seconds, with the total radiation dose being a fraction of that associated with MDCT.

## 2. Case Reports

*Case 1:* 

A 27-year old female patient with an unremarkable medical history was seen for orthodontic evaluation. CBCT was performed for evaluation of skeletal pattern for treatment planning purposes. The patient underwent imaging in Maxillofacial Radiology. CBCT images were acquired using the iCAT™ Imaging system (Imaging Sciences International, Hatfield, PA, USA) using 17 cm × 23 cm field-of-view at 120 kVp and 37 mA, as per imaging protocols set up for orthodontic evaluation. Images were acquired and saved as a DICOM dataset. All data was evaluated using Invivo5 (Anatomage, San Jose, CA, USA). The dataset was evaluated by board-certified oral and maxillofacial radiologists following dental and multiplanar reconstruction. 

**Figure 1:**
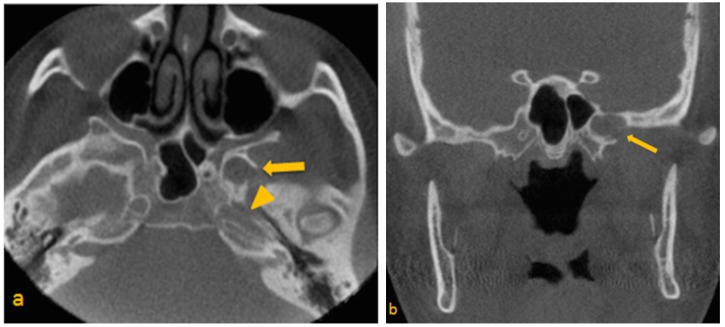

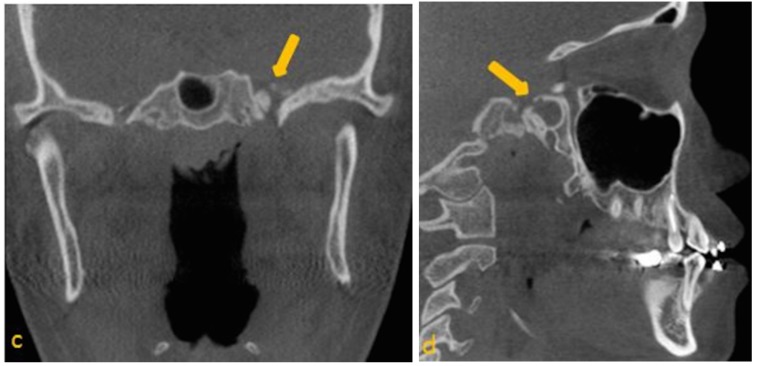
(**a**) Para-axial; (**b,c**) Paracoronal and (**d**) Parasagittal CBCT images through sphenoid Show low-attenuation area in the greater wing of sphenoid on the left side. (a) Para-axial image, depicts suspected disruption of the superior lateral wall of the carotid canal (arrow head); (b) Paracoronal view, note at the close proximity of entity to the vidian canal; (c) Paracoronal image, note at the irregular border of the left foramen ovale and presence of calcification; (d) Parasagittal view, shows extension of entity to the left pterygoid process.

Upon review, a low-attenuation area was noted within the greater wing of the sphenoid on the left, in close proximity to the sphenoid sinus. The borders appeared to be corticated for the most part, with apparent disruption in the inferolateral aspect, where margins were irregular and relatively ill-defined. Foramen rotundum and the vidian canal appeared effaced and difficult to trace in their entirety (Figure 1a–d). Based on radiographic appearance and proximity to critical anatomic entities, a primary differential diagnosis of arrested pneumatization of the sphenoid sinus, fibro-osseous lesion, and arachnoid granulations invading skull base, was made. A larger distance was noted between the pterygoid canal and rotundum. Significant asymmetry of the skull base was noted. Neoplasms were also considered in the differential diagnosis of the feature due to their tendency to be asymptomatic until they invade adjacent structures [9].

Since CBCT only provides three-dimensional (3D) clear images of high-contrast structures and is extremely valuable for evaluating hard tissues of maxillofacial region, it was decided to pursue other advanced imaging to clearly delineate the lesions [10]. CBCT inherently lacks the differential attenuation for soft tissue. Hence, magnetic resonance imaging (MRI) was recommended to evaluate the internal contents and assess the extent of soft tissue involvement including the brain and the meninges [11,12,13]. However, metallic objects, such as orthodontic hardware, cause artifacts from field distortion on head and neck MR images. The patient was therefore advised to have the orthodontic hardware removed to enable her to undergo MR imaging [14].

**Figure 2:**
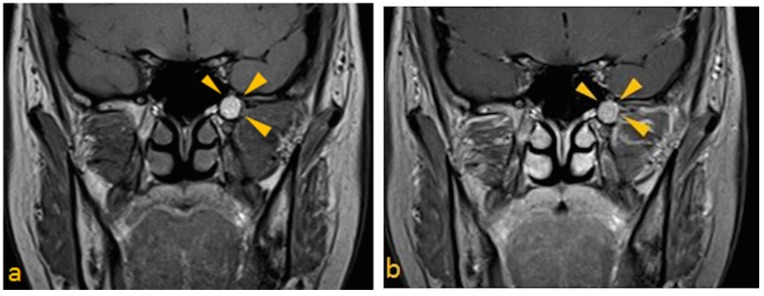

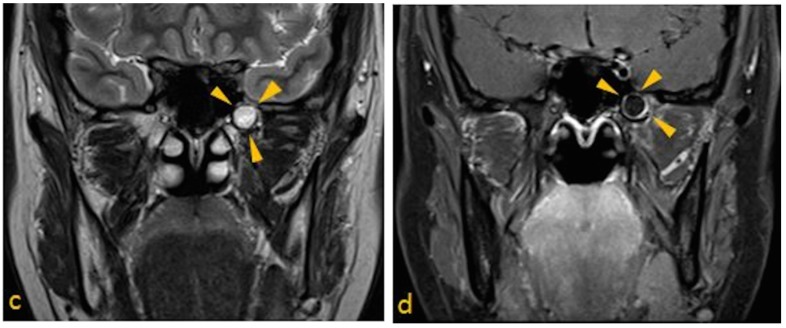
MRI features of arrested pneumatization of sphenoid sinus. (**a**) T1 paracoronal pre-contrast image; (**b**) T1 paracoronal post contrast image; (**c**) T2 paracoronal post gadolinium images, show hyperintense lesion in the left pterygoid process without enhancement; and (**d**) T1 paracoronal post-FS, shows homogeneous loss of signal, consistent with fat.

3T MRI of the head was performed taking care to include the skull base in its entirety. Spin-echo T1- pre- and post-contrast (gadolinium) sequences (slice thickness of 3 mm; TR/TE, 700/10), and spin-echo T2 post-gadolinium data sets were acquired (slice thickness of 3 mm; TR/TE, 3000/101). A hyperintense lesion centered in the left pterygoid process, which measured 10 mm in diameter, was noted, that demonstrated no appreciable enhancement or internal complexity (Figure 2a–c). Spin-echo T1 fat-suppressed images (slice thickness of 3 mm; TR/TE, 838/10) were also obtained to suppress the signal from normal adipose tissue. The sequence revealed homogenous loss of signal, consistent with fat (Figure 2d). The lesion was noted to not involve the contents of foramen ovale and rotundum. Comparison with the CBCT study confirmed that the area demonstrated little evidence of biologic activity. This is most consistent with an area of arrested pneumatization.

*Case 2:* 

A 73-year-old male with history of arthritis, inguinal hernia surgery and malignant bladder cancer presented for implant placement evaluation. The patient was referred to Maxillofacial Radiology for CBCT evaluation using the iCAT 3D Dental Imaging system (Imaging Sciences International, Hatfield, PA, USA) with 16 cm ×10 cm field-of-view (FOV) at 120 kVp and 18 mA. Images were saved in DICOM format and evaluated using Invivo5 by a board-certified maxillofacial radiologist.

**Figure 3:**
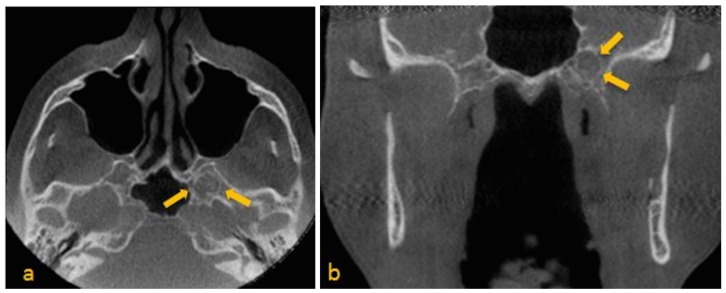
(**a**) Para-axial; (**b**) Paracoronal CBCT images show mixed density area in the left greater wing of the sphenoid adjacent to the sphenoid sinus. Note curvilinear calcifications within this area. (a) Para-axial view, shows suspected disruption of the vidian canal on the left side; (b) Paracoronal view, depicts vidian canal wider than the right side.

Image sequence revealed a well-defined, corticated, ovoid, mixed-density signal in the greater wing of the sphenoid on the left, adjacent to the sphenoid sinus. There were central heterogeneous, high-attenuation entities with signal intensities approximating those of osseous tissue. Possibility of involvement of vidian canal and foramen rotundum could not be ruled out. Based upon the radiographic presentation, an impression of arachnoid granulations was made. There was also evidence of bowing and thinning of the lingual cortical plate at the left mandibular ramus superior to the lingula of the mandibular foramen. Thinning of the facial cortical plate in this region was also noted (Figure 3a,b). Possibility of a benign lesion in the adjacent soft tissue was considered. Based on incidental findings in the study, patient’s medical history, and consensus arrived at by board certified maxillofacial and neuroradiologists, MRI was recommended to further evaluate the lesion of interest.

**Figure 4:**
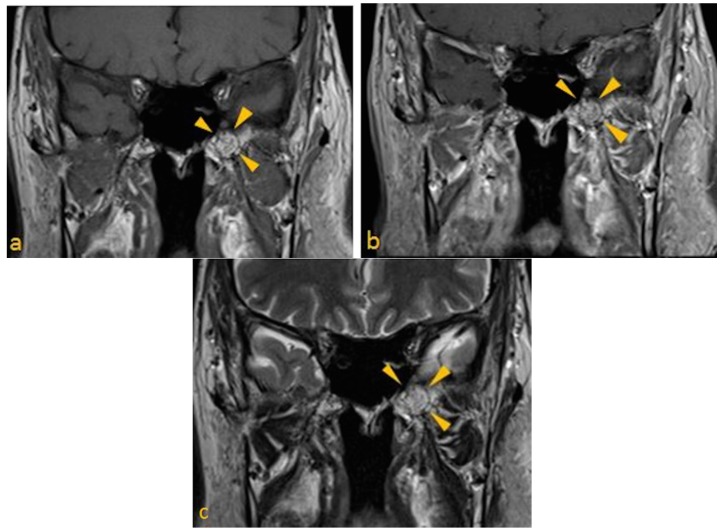
Brain MRI Paracoronal images (**a**) T1 pre-contrast; (**b**) T1 post gadolinium; and (**c**) T2 post gadolinium images, show arrested pneumatization of left sphenoid sinus. Note mild architectural distortion without a focal mass lesion in the marrow of the left pterygoid.

3T MRI of brain was performed. Spin-echo T1- pre- and post-gadolinium images (slice thickness of 3 mm; 31 slices; TR/TE, 635/8.7), and turbo spin echo T2 post-gadolinium image (slice thickness of 3 mm; 31 slices; TR/TE, 5000/102) were obtained (Figure 4a–c). The examination demonstrated mild architectural distortion and heterogeneously high T1 signal intensity (fat), without a focal mass lesion in the marrow of the left pterygoid process. No evidence of foramen ovale and rotundum involvement was noted. These radiographic appearances were related to arrested pneumatization of the left pterygoid process.

## 3. Discussion

There is considerable variation in degree of pneumatization of sphenoid sinus, ranging from minimal to extensive aeration [15]. Sphenoid sinus pneumatization sometimes extends into adjacent bony structures such as the palatine bone, vomer, occipital bone, and ethmoid bones [16]. The common sites of pneumatization are anterior clinoid process and pterygoid process [15]. During the process of normal aeration of sphenoid sinus or other skull base regions, arrested pneumatization may occur [1]. Non-pneumatized or partly pneumatized area of skull base usually contains atypical fatty foci of bone marrow, which appears as a T1 hyperintense signal on MR images. Moreover, fat signal appears on all pulse sequences of MRI post-contrast [1]. Fat-suppressed sequence help to detect adipose tissue by suppressing the high signal of fat and revealing homogenous loss of signal [17].

Diagnosing arrested pneumatization is important in order to distinguish this entity from other skull base abnormalities like fibrous dysplasia, arachnoid granulations, invasive lesions such as acoustic neuroma, glioma, pituitary tumor such as an adenoma, giant cell tumor, chondrosarcoma, metastatic lesions, meningioma, or chordoma [1]. Suggestive radiolographic criteria for arrested pneumatization of sphenoid sinus are defined as:

- Lesion must be non-expansile with sclerotic, well-defined margin, and should be located at a site of normal pneumatization. Evidence of fatty content should be present. Internal curvilinear calcifications should be noted on CT images [1].
- Since, sphenoid sinus is closely related to several vital neurovascular structures such as internal carotid artery and optic nerve, any associated skull-base foramina should remain intact and patent.

Fibrous dysplasia and ossifying fibroma should be distinguished from arrested pneumatization of sphenoid sinus [18]. Fibrous dysplasia is a developmental defect of osseous tissue in which normal bone is replaced by abnormal fibro-osseous tissue [19]. Fibrous dysplasia usually has a ground glass appearance on radiographs. It can cause bony expansion and may compromise neural foramina [20,21].

Ossifying fibroma is a benign neoplasm, which is a unilocuolar, mixed-density lesion that presents with cortical expansion, and usually occurs in the mandible, at times rarely invading the paranasal sinuses [18,22]. Aggressive form of ossifying fibroma invades the paranasal sinuses or maxilla; thus, it is important to distinguish this lesion from arrested pneumatization of sphenoid sinus [22]. 

Chordomas are malignant tumors and should be distinguished from arrested pneumatization [1]. Intracranial chordomas are skull-base tumors that originate from remnants of the primitive notochord. It is described as well-defined, expansile, and destructive soft-tissue mass with no evidence of central foci of fat [1,23].

Chondrosarcomas are a malignant neoplasm of cartilaginous origin [24]. Intracranial chondrosarcomas include 6% of all skull base neoplasms [25]. Majority of them originate in the petro-occipital fissure and occur more laterally, however, they can sometimes be located centrally. It is described as an expansile and osteolytic lesion [23].

Arachnoid granulations serve to drain the cerebrospinal fluid (CSF) and are mainly located in the parasagittal region along the superior sagittal sinus. They may aberrantly be located in the floor of the anterior and middle cranial fossa where they do not drain CSF into the venous system. Therefore, the pressure of CSF will rise, causing bone erosion [26]. It is important to distinguish this lesion from arrested pneumatization of sphenoid sinus because they are usually asymptomatic and may be discovered incidentally. Arachnoid granulations appear hypointense on T1-weighted and hyperintense on the T2-weighted MR images [27]. All of the above features help distinguish them from arrested pneumatization.

## 4. Conclusion

Two cases of incidental findings on the CBCT were reported and were diagnosed as arrested pneumatization of the sphenoid sinus. Dentists should be aware of these incidental findings on CBCT studies. It is important to carefully evaluate the entire volume and not just the region of interest [28].

CBCT imaging of arrested pneumatization of sphenoid sinus reveal important radiographic features that are useful for the differential diagnosis of potential lytic central skull base pathoses. Arrested pneumatization of sphenoid sinus is described as a non-expansile entity with well-circumscribed, sclerotic margins that has curvilinear internal calcifications and internal fatty content. Lesion should localize in a site of common pneumatization of sphenoid sinus. MRI is recommended for more definitive diagnoses. Hence, erosion of the bone adjacent to a lesion on CT, significant enhancement and lack of fat content on MRI should always lead to a diagnosis of more aggressive lesions that need to be explored further.

Features listed in this study must be recognized to prevent unnecessary biopsies or exploratory surgeries thus reducing treatment costs and alleviating anxiety in patients. Occasionally, regions of arrested pneumatization may not demonstrate all these diagnostic criteria. In such cases, periodic follow-up is recommended to establish definitive diagnoses over time, in the absence of clinical signs or symptoms indicative of invasive lesions requiring immediate intervention.
